# A Complicated Course of Pulmonary Tuberculosis With Cavitary Findings and Pleural Effusion

**DOI:** 10.7759/cureus.106192

**Published:** 2026-03-31

**Authors:** Michael W Krakowski, M. Eulalia Brito Vera, Javier López-Royo Díaz de la Peña, Garrett A Perchetti, Ryan Gifford, Roxana Lazarescu

**Affiliations:** 1 Internal Medicine, Touro College of Osteopathic Medicine, New York, USA; 2 Internal Medicine, Francisco de Vitoria University, Madrid, ESP; 3 Internal Medicine, Wyckoff Heights Medical Center, New York, USA

**Keywords:** caseating granulomas, cavitations, mrsa, pleural effusion, tuberculosis

## Abstract

This report describes the case of a 39-year-old male who recently immigrated from Ecuador and presented with recurrent fevers, pleuritic chest pain, and progressive respiratory symptoms initially treated as community-acquired pneumonia. Despite empiric antibiotic therapy, the patient experienced clinical deterioration with rapid accumulation of a left-sided pleural effusion and imaging evidence of a cavitary lung lesion. Pleural fluid analysis demonstrated a lymphocyte-predominant exudative effusion with elevated adenosine deaminase levels, raising suspicion for tuberculous pleuritis despite repeatedly negative acid-fast bacilli smears and cultures, a well-recognized limitation in tuberculous pleuritis given the paucibacillary burden and slow growth of *Mycobacterium tuberculosis*. Definitive diagnosis was established via video-assisted thoracoscopic surgery, with pleural biopsy revealing caseating granulomas consistent with tuberculosis (TB), with the clinical course further complicated by methicillin-resistant *Staphylococcus aureus *empyema. The patient was successfully treated with surgical drainage and guideline-directed anti-tuberculous therapy, resulting in clinical improvement. This case underscores the diagnostic challenges of extrapulmonary TB in non-endemic regions and highlights the importance of maintaining a high index of suspicion in patients from endemic areas, even when initial microbiologic testing is negative.

## Introduction

Tuberculosis (TB) is a human infectious disease caused by *Mycobacterium tuberculosis*. It primarily affects the lungs, known as pulmonary TB, though extrapulmonary involvement occurs in one-third of patients [1]. Globally, TB is a leading cause of death due to infectious diseases, accounting for an estimated 1.5 million deaths annually. It is estimated that nearly two billion people worldwide are infected with *M. tuberculosis*, the majority of whom have latent TB infection rather than active disease [1]. It is an airborne disease, which is transmitted by droplet nuclei produced by an active TB patient [1]. The clinical presentation of pulmonary TB includes unilateral pleuritic chest pain (75% of patients), cough (70% of patients), fever (85%), night sweats (50%), dyspnea (50%), and weight loss (25-85%) [2].

One complication of pulmonary TB is pleural effusion [3]. The incidence of pleural effusion in TB disease varies geographically, ranging from 3% to 5% in non-endemic areas to up to 30% in endemic areas [2]. While this condition more frequently affects younger patients globally, it tends to present in older populations in non-endemic settings [2]. The pathophysiology involves a combination of a delayed hypersensitivity reaction and direct pleural infection. Diagnostic evaluation often includes thoracentesis with measurement of adenosine deaminase (ADA) and interferon-gamma levels in pleural fluid, which can support the diagnosis. While mycobacterial culture remains the gold standard due to its high specificity (>97%), results may be delayed or initially negative. In such cases, lung or pleural biopsy may be necessary to demonstrate caseating granulomas or confirm infection when less invasive testing is inconclusive [2].

Despite its global burden, TB may be under-recognized in developed, non-endemic regions, where clinicians are less likely to encounter the disease routinely. This diagnostic challenge is particularly relevant in the United States, where TB disproportionately affects individuals born outside the country, who account for the majority of new cases despite representing a smaller proportion of the population. Prior studies have demonstrated that targeted post-arrival evaluation of at-risk immigrants can be highly effective in identifying TB infection. However, gaps in screening completion and treatment of latent TB infection (LTBI) remain significant challenges [4]. These disparities highlight the importance of incorporating epidemiologic risk factors, such as recent immigration from endemic regions, into early clinical decision-making.

In this context, patients presenting with persistent respiratory symptoms or pleural effusion, particularly those with epidemiologic risk factors or failure to respond to initial empiric therapy, require a high index of suspicion for TB to avoid delays in diagnosis and management. Although most tuberculous pleural effusions may resolve spontaneously, treatment remains essential, as up to 65% of patients may progress to active pulmonary TB within two years if left untreated [2]. Additionally, untreated TB carries a mortality rate of approximately 50-65% within five years [1].

## Case presentation

A 39-year-old male with no significant past medical history, originally from Ecuador and having immigrated to the United States one year prior, presented to the emergency department (ED) with a three-day history of flu-like symptoms, including cough, fever, vomiting, diarrhea, body aches, and headache. His vaccination history was unknown. The patient is an on-and-off smoker but denied any known comorbidities. On presentation, he was febrile to 39.4°C, tachycardic at 112 bpm, and normotensive at 112/72 mmHg. Physical examination revealed constant aching chest and back pain rated at 5 out of 10. Initial infectious workup, including COVID-19 RT-PCR, influenza A/B, and RSV (Adult4Plex) testing, was negative. Initial chest X-ray demonstrated a left-sided basilar consolidation with associated pleural effusion. The patient received 2 L of lactated Ringer's solution and a single dose of IV ceftriaxone in the ED for empiric treatment of suspected community-acquired pneumonia (CAP) and was discharged home with antipyretics (acetaminophen) and instructions to return if symptoms worsened. Although initially managed as CAP, his subsequent clinical course revealed a unifying diagnosis of active pulmonary TB with associated tuberculous pleuritis, complicated by secondary methicillin-resistant *Staphylococcus aureus *(MRSA) empyema.

**Figure 1:**
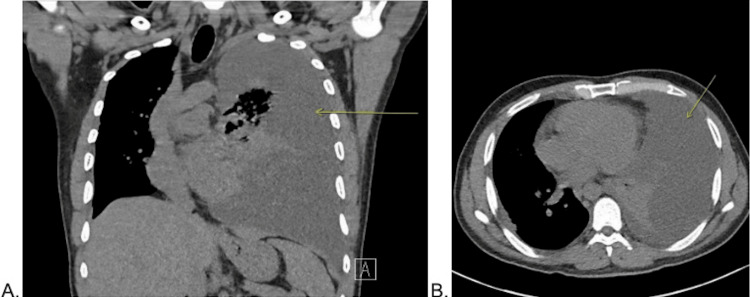
CT of the chest CT of the chest, coronal cross-section (A) and axial cross-section (B), indicating a large left-sided pleural effusion (arrow).

The patient returned to the ED four days later with persistent fever, fatigue, worsening upper respiratory symptoms, and inability to tolerate oral intake due to nausea and six episodes of vomiting since initial discharge. He reported difficulty sleeping and continued fevers despite the use of acetaminophen. On evaluation, his temperature was 38.2°C, with a heart rate of 123 bpm and a respiratory rate of 22 breaths/min, meeting three out of the four Systemic Inflammatory Response Syndrome (SIRS) criteria [5]. He was admitted for sepsis secondary to CAP with a suspected complicated parapneumonic effusion and started on IV ceftriaxone and azithromycin. A CT of the chest revealed a significantly increased left-sided pleural effusion with near-complete opacification of the left hemithorax, superimposed left lung atelectasis, and concern for a cavitary lung lesion (Figure 1). Given his recent immigration from a TB-endemic region and concerning radiographic findings, he was placed in airborne isolation for suspected pulmonary TB. Three sputum samples were collected for acid-fast bacilli (AFB) smear and culture, and a QuantiFERON-TB Gold test was ordered. Daily chest X-rays were performed throughout the hospital course to monitor the progression of the effusion and consolidation.

A left-sided pigtail catheter was placed the same day, draining 1.75 L of serous pleural fluid. Pleural fluid was sent for cytologic and microbiologic analysis, including AFB smear and culture and ADA. Pleural fluid analysis showed a cloudy exudative effusion by Light's criteria with a high pleura/serum lactate dehydrogenase (LDH), white blood cell (WBC) of 250,000 cells/µL with 66% lymphocytes, and ADA of 59 IU/L (Table 1) [6]. These findings, though not diagnostic, raised high clinical suspicion for tuberculous pleuritis. Initial AFB smears from both sputum and pleural fluid were negative.

**Table 1:** Pleural fluid analysis

Pleural Fluid Parameter	Patient Value	Normal Range
Appearance	Cloudy	Clear
Color	Yellow	Clear to pale yellow
WBC (/µL)	250,000	<1,000
RBC (/µL)	889	<10,000
Neutrophils (%)	26%	<25%
Lymphocytes (%)	66%	<50%
Monocytes (%)	8%	Variable
Adenosine Deaminase (U/L)	56.5	<40
Other Cells	Mesothelial cells	Few mesothelial cells

On hospital day five, the patient consented to and underwent video-assisted thoracoscopic surgery (VATS) with pleural biopsy and decortication. Intraoperative findings included diffuse inflammation of the pleural cavity with multiloculated empyema and copious pleural rind along the lower lobe of the left lung and the diaphragm. Specimens were obtained from the left lung pleura and pleural effusion for Gram stain, AFB culture, and histopathology. A frozen section analysis of pleural tissue revealed caseating granulomas consistent with TB. Given the intraoperative diagnosis of TB, the decortication was aborted. Three chest tubes (20 Fr) were placed in the left hemithorax: an angled chest tube superior to the diaphragm, a middle tube directed posteriorly, and a posterior-most tube angled anteriorly toward the apex.

Postoperatively, the patient was admitted to the surgical ICU for monitoring and pain control. He began RIPE (rifampin, isoniazid, pyrazinamide, ethambutol) therapy, along with pyridoxine (vitamin B6) supplementation. Gram stain and culture from the pleural fluid grew MRSA, prompting the initiation of IV linezolid 600 mg BID for a planned seven-day course of MRSA coverage. QuantiFERON-TB testing returned positive. Despite the TB-positive QuantiFERON, RT-PCR, and caseating granulomas seen on frozen sections, AFB cultures from sputum and pleural samples remained negative. The infectious disease team recommended a full course of RIPE therapy with B6 for two months, followed by continuation of isoniazid and rifampin with B6 for an additional four to seven months, depending on clinical response.

The patient was closely monitored and showed steady improvement. He was transferred to the intermediate care unit (IMCU) on hospital day nine. The chest tubes were removed on hospital day 10. The patient remained afebrile, demonstrated improved pain control, and tolerated oral intake.

On hospital day 13, the patient was transferred from the IMCU to the general medical floor, where isolation precautions were maintained. He remained clinically stable. Chest X-ray showed decreased but moderate left pleural effusion, with improved aeration of the left lung base and resolution of mild airspace disease. On hospital day 15, the patient on airborne precautions was discharged home with a 30-tablet prescription of RIPE therapy and pyridoxine, with plans to continue therapy through his local TB center. He was informed that isoniazid was on temporary back order and was instructed to resume it once available.

## Discussion

Tuberculous pleural effusion represents one of the most common extrapulmonary manifestations of TB associated with *M. tuberculosis *antigens in the pleural space [7]. The presence of both a cavitary lesion and pleural effusion, as in this case, suggests extensive pulmonary involvement and a more complicated disease course. Cavitation reflects active parenchymal destruction and higher bacillary load, which may increase transmission risk and delay response to therapy [8].

Pleural effusions in TB can be exudative and lymphocyte-predominant, often with elevated ADA levels. ADA levels above 40 IU/L are considered strongly suggestive of tuberculous pleural effusion, as seen in this patient (59 IU/L) [6]. Although AFB smears and cultures are frequently negative in pleural fluid, tissue biopsy remains the diagnostic gold standard, demonstrating caseating granulomas or positive cultures for *M. tuberculosis *[9].

The patient's disease course was further complicated by MRSA co-infection, highlighting the importance of considering secondary bacterial infection in patients with TB who present with empyema or fail to improve with standard therapy [10]. MRSA superinfection may arise in areas of damaged lung parenchyma or within complex pleural effusions and has been associated with increased morbidity, prolonged hospitalization, and the need for procedural intervention [10]. In this case, persistent symptoms and progressive pleural disease despite initial empiric antibiotic therapy suggested a more complex underlying process, prompting further diagnostic evaluation and intervention. The decision to perform VATS for decortication and biopsy allowed for both diagnostic clarification and effective drainage of the complicated effusion [11]. Targeted antimicrobial therapy with linezolid, in conjunction with drainage and anti-tuberculous treatment, was essential for clinical improvement. Post-surgical recovery and subsequent RIPE therapy further illustrate the benefit of a combined surgical and medical approach in managing complicated tuberculous pleural effusions with bacterial superinfection.

Cavitary TB lesions may prolong infectivity and pose a higher relapse risk if therapy is interrupted or if drug resistance develops [12]. Therefore, strict adherence to RIPE therapy and drug-susceptibility testing remains an essential component of management, particularly among immigrants from endemic regions where resistant strains are prevalent.

## Conclusions

This case illustrates the clinical course of pulmonary TB complicated by cavitary lesions and pleural effusion. Despite a delayed diagnosis of TB and *S. aureus *bacterial co-infection, a combination of surgical intervention, pleural biopsy, and guideline-based RIPE therapy achieved favorable outcomes. Early suspicion, comprehensive diagnostic evaluation, and multidisciplinary management are key to optimizing prognosis in complicated TB presentations. Even in low-incidence settings, such as the United States, it is critical to maintain clinical suspicion for the diagnosis of TB in order to minimize complications and improve treatment outcomes.
